# Slow rewarming after hypothermia does not ameliorate white matter injury after hypoxia-ischemia in near-term fetal sheep

**DOI:** 10.1038/s41390-024-03332-y

**Published:** 2024-08-05

**Authors:** Alice McDouall, Kelly Q. Zhou, Anthony Davies, Guido Wassink, Timothy L. M. Jones, Laura Bennet, Alistair J. Gunn, Joanne O. Davidson

**Affiliations:** Department of Physiology, The University of Auckland, Auckland, New Zealand

## Abstract

**Background:**

The optimal rate to rewarm infants after therapeutic hypothermia is unclear. In this study we examined whether slow rewarming after 72 h of hypothermia would attenuate white matter injury.

**Methods:**

Near-term fetal sheep received sham occlusion (*n* = 8) or cerebral ischemia for 30 min, followed by normothermia (*n* = 7) or hypothermia from 3–72 h, with either spontaneous fast rewarming (*n* = 8) within 1 h, or slow rewarming at ~0.5 °C/h (*n* = 8) over 10 h. Fetuses were euthanized 7 days later.

**Results:**

Ischemia was associated with loss of total and mature oligodendrocytes, reduced expression of myelin proteins and induction of microglia and astrocytes, compared with sham controls (*P* < 0.05). Both hypothermia protocols were associated with a significant increase in numbers of total and mature oligodendrocytes, area fraction of myelin proteins and reduced numbers of microglia and astrocytes, compared with ischemia-normothermia (*P* < 0.05). There was no difference in the number of oligodendrocytes, microglia or astrocytes or expression of myelin proteins between fast and slow rewarming after hypothermia.

**Conclusion:**

The rate of rewarming after a clinically relevant duration of hypothermia had no apparent effect on white matter protection by hypothermia after cerebral ischemia in near-term fetal sheep.

**Impact:**

- Persistent white matter injury is a major contributor to long-term disability after neonatal encephalopathy despite treatment with therapeutic hypothermia.
- The optimal rate to rewarm infants after therapeutic hypothermia is unclear; current protocols were developed on a precautionary basis.
- We now show that slow rewarming at 0.5 °C/h did not improve histological white matter injury compared with rapid spontaneous rewarming after a clinically established duration of hypothermia in near-term fetal sheep.

## Introduction

White matter injury is common after moderate to severe neonatal hypoxic-ischemic encephalopathy (HIE).^1–3^ Neonatal white matter injury is highly associated with later communication and behavioral problems, visual impairments, and seizures, although interestingly, not with adverse motor outcomes.^4^ On magnetic resonance imaging (MRI), combining the scores for injury severity of the basal-ganglia/thalami with that for white matter, was the greatest predictor of adverse outcomes at 2-years in infants treated with therapeutic hypothermia.^5^

Therapeutic hypothermia significantly reduces white matter abnormalities in infants with HIE.^6–8^ However, despite treatment with hypothermia, there can still be prolonged changes within the white matter. For example, infants treated with hypothermia had widespread microstructural abnormalities within white matter areas within the first week of life.^9^ Infants who had lower fractional anisotropy values, which can be due to reduced fiber density, area or myelination in the first week of life, had higher risks of unfavorable outcomes including death and severe cerebral palsy at 24 months of age.^9^ Further, children who did not develop cerebral palsy after being treated with hypothermia had significantly lower fractional anisotropy values compared with age matched controls at 6–8 years of age.^10^ Together, this suggests that microstructural differences in white matter persist throughout childhood, and that despite treatment with hypothermia, infants with HIE may have impaired neurodevelopment.

Given the persistent abnormalities within the white matter despite hypothermia, there is a need to optimize the current protocols for hypothermia. Extensive pre-clinical trials have shown that hypothermia started as early as possible before 6 h, and continued for 72 h, results in optimal neuroprotection.^11–14^ However, there is limited evidence for the optimal rate to rewarm after hypothermia.^15,16^ Hypothermia reduces oxidative stress, excitotoxin release, programmed cell death and inflammatory responses, all of which could theoretically be reactivated during rewarming.^17^

In near-term fetal sheep, slow rewarming over 24 h after 48 h of hypothermia following 30 min of hypoxia-ischemia (HI), was not associated with significant improvement in white matter injury compared with rapid spontaneous rewarming within 1 h following 72 h of hypothermia.^18^ Strikingly, only rapid rewarming following 72 h of hypothermia was associated with significant attenuation of the loss of mature oligodendrocytes compared with normothermia. Furthermore, slower rewarming did not further improve the neurophysiological recovery or cortical neuronal survival compared with fast rewarming.^19^ In neonatal pigs there was no significant difference in caspase 3 activation between slower (0.5 °C/h) and faster rewarming (4 °C/h) after 18 h of hypothermia.^20^ However, it should be noted that interpretation of the effect of the rate of rewarming in both the pig and fetal sheep studies may be confounded by the preceding suboptimal durations of hypothermia. In previous pre-clinical studies 72 h provides better protection of white and gray matter than shorter durations.^11,21,22^ Therefore, it is still unclear whether slow rewarming improves white matter recovery after a clinically relevant duration of hypothermia.

The aim of the current study was to determine if slow rewarming at ~0.5 °C per hour compared with spontaneous, fast rewarming over ~1 h, after an optimal duration of hypothermia, would improve recovery of the white matter, including oligodendrocyte survival, myelination, and inflammation after global cerebral ischemia in the term-equivalent fetal sheep.

## Methods

All procedures were approved by the Animal Ethics Committee of The University of Auckland under the New Zealand Animal Welfare Act, and the Code of Ethical Conduct for animals in research, established by the Ministry of Primary Industries, Government of New Zealand. The experiment has been reported in compliance with the ARRIVE guidelines.^23^

31 Romney ewes were acclimatized for 7 days prior to surgery in metabolic crates in a holding room with a temperature of 16 ± 1 °C and 50 ± 10% humidity on a 12 h light/dark cycle. Food but not water was restricted 18 h before surgery. At 125–126 days gestation ewes underwent sterile surgery for instrumentation of both male and female Romney/Suffolk fetuses. Ewes were given an intramuscular injection of long acting oxytetracycline at 1 mL/10 kg (20 mg/kg, Phoenix Pharm, Auckland, New Zealand) for prophylaxis 30 min prior to surgery. Anesthesia was induced by intravenous propofol (5 mg/kg; AstraZeneca Limited, Auckland, New Zealand) into the brachial vein and maintained with 2–3% isoflurane (Bomac Animal Health, New South Wales, Australia) in oxygen after intubation until the end of the surgery. During surgery, maternal fluid was maintained by a constant infusion of Plasma-Lyte 148 (Baxter, Auckland, New Zealand) into the brachial vein. The depth of anesthesia, maternal blood pressure and heart rate were monitored by trained anesthetic staff during surgery.

Surgery was performed using aseptic techniques. A skin incision on the maternal midline over the abdomen was made, followed by a laparotomy along the linea alba. An incision of the uterus allowed the fetus to be exposed. Fetal brachial arteries were catheterized with saline filled customized polyvinyl catheters (SteriHealth, Dandenong South, Victoria, Australia) in order to measure mean arterial blood pressure. An additional catheter was attached to the skin of the fetal shoulder to measure amniotic pressure. A pair of electrodes (Cooner Wire Co., Chatsworth, California, USA) was attached subcutaneously onto the right shoulder and the left fifth intercostal space for monitoring of fetal heart rate. Vertebral-occipital anastomoses were ligated to prevent vertebral blood supply to the carotid arteries. In-house inflatable occluder cuffs made of silicon tubing (Degania Silicone, Kibbutz Degania Bet, Israel) were placed around both carotid arteries to enable the induction of cerebral ischemia. An ultrasound flow probe (3 S type, Transonic Systems, Ithaca, New York, USA) was placed inferiorly to the carotid occluders to measure cerebral blood flow. Two pairs of electrodes (Cooner Wire) were placed on the dura over the parasagittal parietal cortex (10 and 20 mm anterior to lambda and 10 mm lateral of the medial longitudinal fissure) to measure the electroencephalogram activity. Electrodes were secured in place with cyanoacrylate glue, with a reference earth wire sewn over the fetal occiput. A third pair of electrodes were placed 5 mm laterally between the two EEG electrodes (Cooner Wire) for measurement of cerebral impedance. A pair of electrodes were sewn into the nuchal muscle to measure electromyogram activity. Two thermistors (Incu-Temp-1; Mallinckrodt Medical, St. Louis, Missouri, USA) were placed over the parasagittal dura ( ~ 30 mm anterior to lambda) and within the esophagus to measure extradural and core temperatures. A cooling cap made in-house from silicon tubing (3 × 6 mm, Degania Silicone) was secured to the fetal head for induction of hypothermia.

The fetus was returned to the uterus and the amniotic fluid lost during surgery was replaced with sterile 0.9% saline. The uterus was closed and gentamicin (80 mg, Pharmacia and Upjohn, Rydalmere, New South Wales, Australia) was administered into the amniotic sac through the amniotic catheter. All fetal leads were exteriorized through the maternal flank. The maternal incision in the linea alba and skin were repaired and 10 mL 0.5% bupivacaine plus adrenaline (AstraZeneca Ltd) was injected into the skin. A catheter was placed into the maternal long saphenous vein for post-operative care and euthanasia.

Following surgery, sheep were housed with other sheep in individual metabolic cages with access to food and water *ad libitum*. Rooms were at a constant temperature of 16 ± 1 °C and 50 ± 10% humidity on a 12 h light/dark cycle. The ewe received benzylpenicillin (600 mg, Novartis Ltd, Auckland, New Zealand) for 4 days and gentamicin (80 mg, Novartis Ltd) for 2 days through the maternal vein catheter. Fetal catheters were continuously infused with heparinized saline (20 U/mL at 0.2 mL/h). Daily arterial blood samples were taken from the fetus to monitor for health, blood gas, base excess (ABL800 Flex Analyzer, Radiometer, Auckland, New Zealand), glucose, and lactate measurements (YSI model 2300, Yellow Springs, Ohio, USA).

### Experimental protocols

**Fig. 1:**
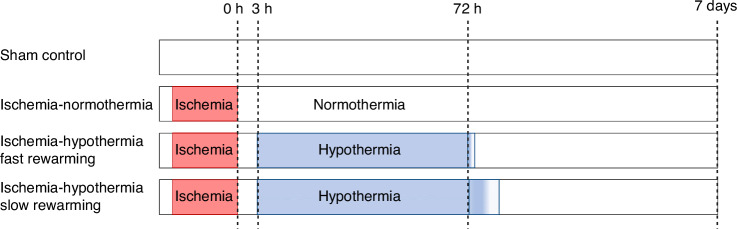
Schematic diagram of experimental protocol. Created with Biorender.com.

Prior to experimentation, instrumented fetuses were randomized using an online random number generator to sham control (*n* = 8), ischemia-normothermia (*n* = 7), ischemia-hypothermia fast rewarming (*n* = 8) or ischemia-hypothermia slow rewarming (*n* = 8, Fig. 1). At 129 ± 1 day gestation, cerebral ischemia was induced in all fetuses apart from sham control, by inflation of the carotid artery occluders with sterile saline for 30 min.

Cooling began 3 h after the end of cerebral ischemia and continued until 72 h, by attaching the cooling coils to a pump circulating cold water. Targeted extradural temperatures for the first 2 h were 31–33 °C to align with previous studies.^13,24^ No water was circulated through the cooling coils for sham control and ischemia-normothermia fetuses. For the ischemia-hypothermia fast rewarming group, the cooling machine was turned off at 72 h and fetuses were allowed to rewarm spontaneously. For the ischemia-hypothermia slow rewarming group, the fetuses were progressively rewarmed over 10 h, with a computer-controlled linear increase in bath temperature up to 39 °C.

7 days after cerebral ischemia, ewes and fetuses were euthanized by overdose of sodium pentobarbitone (9 g i.v. to the ewe; Pentobarb 300; Chemstock, Christchurch, New Zealand). Fetal brains were perfusion fixed via cannulation of both carotid arteries with isotonic heparinized saline, followed by 10% phosphate-buffered formalin (Global Science, Auckland, New Zealand). Brain tissue was post-fixed by immersion in 10% phosphate buffered formalin for 1 week, and then embedded in paraffin.

### Immunohistochemistry

Coronal slices were cut using a microtome (10 µm thick, Leica Jung RM2035, Wetzlar, Germany) at the level of the mid striatum and dorsal hippocampus. Slices were mounted on poly-L-lysine (Sigma-Aldrich, St Louis, Missouri, USA) coated slides and oven dried. Slides were dewaxed in xylene and rehydrated in decreasing concentrations of ethanol, and then washed with phosphate-buffered saline (PBS). Slides were placed in citrate buffer and placed in a pressure cooker (2100 Antigen Retriever, Aptum Biologics Ltd., Southampton, England) for antigen retrieval. To prevent endogenous peroxidase activity, slides were incubated in 1% H_2_O_2_ in methanol (or PBS for Olig-2 and CC1 labeling) for 30 min. Slides were blocked in 3% normal goat serum (NGS) in PBS for 1 h at room temperature. Sections were incubated with primary antibodies (1:200) with 3% NGS in PBS overnight at 4 °C. These included mouse anti-ionized calcium-binding adapter molecule 1 (Iba-1, ab15690, Abcam, Cambridge, England); mouse anti-myelin basic protein (MBP, MAB381, Millipore, Burlington, Massachusetts, USA), mouse anti-2′,3′-Cyclic-nucleotide 3′-phosphodiesterase (CNPase, AB6319, Abcam) as a marker of immature/mature oligodendrocytes; mouse anti-APC antibody (CC-1, AB16794, Abcam); mouse anti-oligodendrocyte lineage transcription factor 2 (Olig-2, AB236540, Abcam) and rabbit anti-glial-fibrillary-acidic protein (GFAP, AB68428, Abcam). Sections were then incubated with secondary antibodies (1:200) with 3% NGS in PBS overnight at 4 °C. Secondary antibodies were either biotinylated goat anti-mouse (BA-9200, Vector Laboratories, Burlingame, CA, USA) or anti-rabbit (BA-1000, Vector Laboratories) as appropriate. Slides were then incubated in ExtrAvidin® (1:200, Sigma-Aldrich Pty. Ltd.) in PBS for 2 h at room temperature.

Diaminobenzidine tetrachloride (Sigma-Aldrich Pty. Ltd) was added to slides for visualization of positive labeling, with the reaction stopped by washing the slide in PBS. Sections were dehydrated in increasing concentrations of ethanol and then placed in xylene, prior to mounting with DPX medium (Sigma-Aldrich Pty. Ltd).

### Imaging and analysis

An investigator blinded to the treatment groups imaged two slides per antibody by bright field microscopy on a Nikon Eclipse 80i (Scitech Ltd, Preston, Victoria, Australia) at 20x magnification. Images were taken in the intragyral white matter tract of the first (IGWM1) and second parasagittal (IGWM2) gyrus and the periventricular white matter (PVWM) from both hemispheres. Numbers of positively labeled cells were quantified by manual counts using ImageJ software (National Institutes of Health, USA). For Iba-1 microglia labeling, both ramified and ameboid microglia were included. For cleaved caspase 3^+^ apoptotic cell labeling, positively-labeled cells, with a clear apoptotic morphology were included. Area fraction for MBP, CNPase and GFAP positive labeling were performed using the Auto threshold settings on ImageJ (National Institutes of Health).

### Statistics

Based on the variance in oligodendrocyte loss after 30 min of carotid artery occlusion in a previous study,^25^ a sample size of *n* = 6/group was estimated to have 90% power to detect a 10% reduction or more in oligodendrocyte counts in the IGWM1. All statistical analysis was performed on SPSS (Version 28, SPSS Inc., Chicago, Illinois, USA). The normality of data distribution was tested using the Shapiro-Wilk test. Differences in sex were analyzed using the Fishers exact test. All outcomes were analyzed by one-way analysis of variance (ANOVA) followed by the Tukey post hoc test or Kruskal-Wallis test for non-parametric data. Statistical significance was reported when *P* < 0.05. All values are expressed as the mean ± standard deviation.

## Results

### Fetal demographics

There was no significant difference in sex between the groups (Table 1, *P* = 0.786). There were no significant differences in body weight between the groups (Table 1, *P* = 0.72). Sham controls had significantly higher brain weight than the ischemia-normothermia group (*P* = 0.04).

**Table 1:** Demographic data at 7 days after 30 min of cerebral ischemia in near term fetal sheep.

Group	Sex (M/F)	Body weight (g)	Brain weight (g)
Sham control	5 M, 3 F	5206 ± 926	49 ± 6
Ischemia-normothermia	3 M, 3 F, 1 U	4584 ± 954	39 ± 3*
Ischemia-hypothermia fast rewarming	3 M, 5 F	4679 ± 433	45 ± 4
Ischemia-hypothermia slow rewarming	4 M, 4 F	4846 ± 775	44 ± 5

Sham control, *n* = 8; ischemia-normothermia, *n* = 7; ischemia-hypothermia fast rewarming, *n* = 8; ischemia-hypothermia slow rewarming, *n* = 8. Data are mean ± standard deviation *P < 0.05 vs. sham control.

*M* male, *F* female, *U* unknown.

### Temperature

Induction of hypothermia was associated with a significant decrease in extradural temperatures in both the ischemia-hypothermia rewarming groups compared with sham control and ischemia-normothermia (*P* < 0.001 ischemia-hypothermia fast rewarming vs. sham control and ischemia-normothermia, *P* < 0.001 ischemia-hypothermia slow rewarming vs. sham control and ischemia-normothermia). In the ischemia-hypothermia fast rewarming group hypothermia resulted in an average decrease in temperature of 5.1 ± 0.9 °C compared with pre-hypothermia temperatures, to an average of 34.4 ± 1.0 °C (Supplementary Fig. 1). In the ischemia-hypothermia slow rewarming group hypothermia resulted in an average decrease in temperature of 5.3 ± 0.9 °C compared with pre-hypothermia temperatures, to an average of 34.5 ± 0.9 °C. During hypothermia there was no significant difference in extradural temperatures between ischemia-hypothermia fast rewarming and ischemia-hypothermia slow rewarming groups (*P* = 1.0). In the ischemia-hypothermia fast rewarming group extradural temperature returned to baseline normothermia temperatures by an average of 44 ± 13 min. In the ischemia-hypothermia slow rewarming group extradural temperatures returned to baseline within 10 h at an average rate of 0.45 ± 0.20 °C/h.

Hypothermia was associated with a significant decrease in esophageal temperature in both the ischemia-hypothermia groups compared with sham control and ischemia-normothermia (*P* < 0.001 ischemia-hypothermia fast rewarming vs. sham control and ischemia-normothermia, *P* < 0.001 ischemia-hypothermia slow rewarming vs. sham control and ischemia-normothermia). In the ischemia-hypothermia fast rewarming group, hypothermia resulted in an average decrease in temperatures of 1.2 ± 0.2 °C compared with pre-hypothermia temperatures, to an average of 38.1 ± 0.3 °C. In the ischemia-hypothermia slow rewarming group hypothermia resulted in an average decrease in temperatures of 1.5 ± 0.4 °C compared with pre-hypothermia temperatures, to an average of 38.2 ± 0.4 °C. During hypothermia there was no significant difference in esophageal temperatures between ischemia-hypothermia fast rewarming and ischemia-hypothermia slow rewarming (*P* = 0.994).

### Immunohistochemistry

**Fig. 2:**
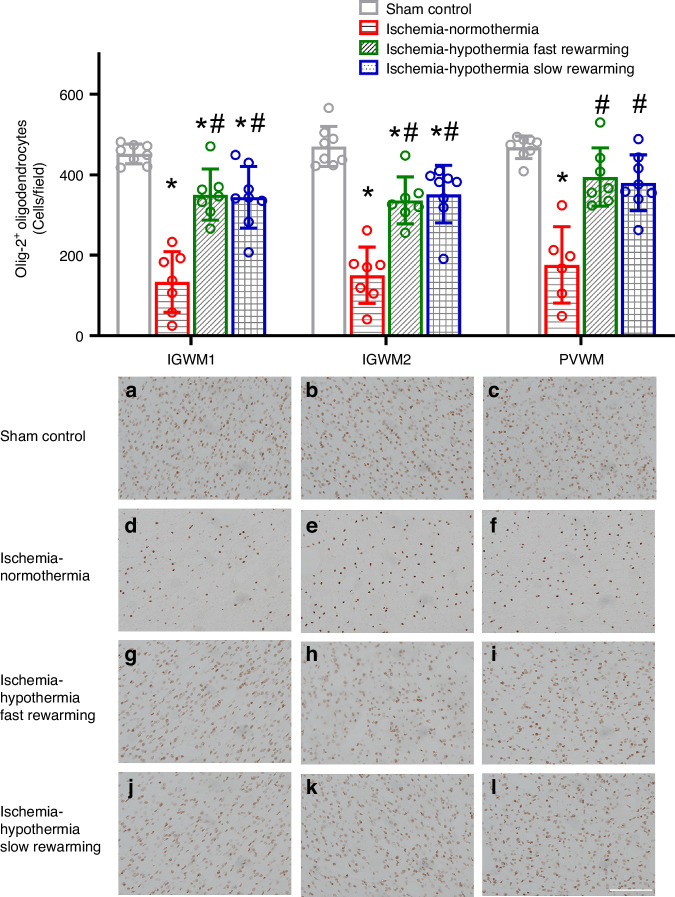
Olig-2^+^ oligodendrocyte number and photomicrographs in the IGWM1, IGWM2, and PVWM in the sham control, ischemia-normothermia, ischemia-hypothermia fast rewarming and ischemia-hypothermia slow rewarming groups 7 days post HI. (Top) Olig-2^+^ oligodendrocyte number within the IGWM1, IGWM2, and PVWM regions. (Bottom) representative photomicrographs of Olig-2^+^ in the IGWM1 (**a**, **d**, **g**, **j**), IGWM2 (**b**, **e**, **h**, **k**), PVWM (**c**, **f**, **i**, **l**). Scale bar in *L* = 100 µm. Sham control, *n* = 8; ischemia-normothermia, *n* = 7; ischemia-hypothermia fast rewarming, *n* = 7; and ischemia-hypothermia slow rewarming groups *n* = 8. Data are mean ± standard deviation. * *P* < 0.05 vs. sham control, # *P* < 0.05 vs. ischemia-normothermia.

Ischemia was associated with a significant reduction in the number of Olig-2^+^ oligodendrocytes in the IGWM1, IGWM2, and PVWM compared with sham control (Fig. 2, *P* < 0.001 for all regions). Both hypothermia protocols were associated with significant preservation in the number of Olig-2^+^ oligodendrocytes compared with ischemia-normothermia in the IGWM1, IGWM2, and PVWM (*P* < 0.001 for all regions). However, this was an intermediate effect in the IGWM1 and IGWM2 with oligodendrocyte numbers remaining significantly less than sham control in these areas (*P* = 0.023, *P* = 0.011 sham control vs. ischemia-hypothermia fast rewarming, and *P* = 0.011, *P* = 0.004 sham control vs. ischemia-hypothermia slow rewarming, in the IGWM1 and IGWM2 respectively). There was no significant difference in the numbers of Olig-2^+^ oligodendrocytes between ischemia-hypothermia fast rewarming and ischemia-hypothermia slow rewarming (*P* = 0.997, *P* = 0.964, *P* = 0.977, in the IGWM1, IGWM2 and PVWM respectively).

**Fig. 3:**
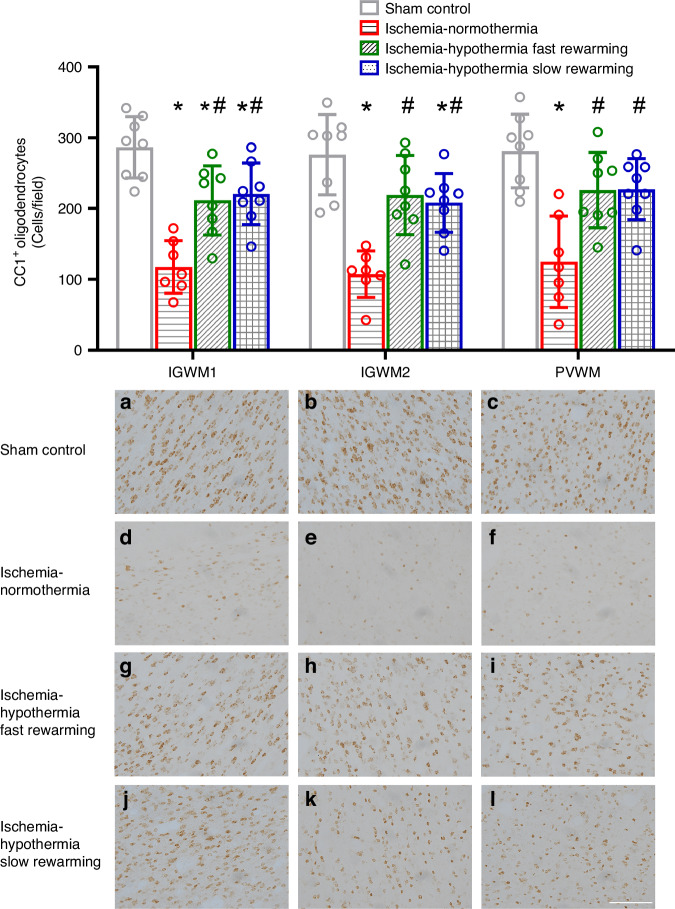
CC1^+^ oligodendrocyte number and photomicrographs in the IGWM1, IGWM2, and PVWM in the sham control, ischemia-normothermia, ischemia-hypothermia fast rewarming and ischemia-hypothermia slow rewarming groups 7 days post HI. (Top) CC1^+^ oligodendrocyte number within the IGWM1, IGWM2, and PVWM regions. (Bottom) representative photomicrographs of CC1^+^ in the IGWM1 (**a**, **d**, **g**, **j**), IGWM2 (**b**, **e**, **h**, **k**), PVWM (**c**, **f**, **i**, **l**). Scale bar in *L* = 100 µm. Sham control, *n* = 8; ischemia-normothermia, *n* = 7; ischemia-hypothermia fast rewarming, *n* = 8 and ischemia-hypothermia slow rewarming groups *n* = 8. Data are mean ± standard deviation. * *P* < 0.05 vs. sham control, # *P* < 0.05 vs. ischemia-normothermia.

Ischemia was associated with a significant reduction in the number of CC1^+^ mature oligodendrocytes in the IGWM1, IGWM2, and PVWM compared with sham control (Fig. 3, *P* < 0.001 for all regions). Hypothermia was associated with a significant preservation of CC1^+^ mature oligodendrocytes in all regions compared with ischemia-normothermia (*P* = 0.002, *P* < 0.001, *P* = 0.006 ischemia-hypothermia fast rewarming vs. ischemia-normothermia, and *P* < 0.001, *P* = 0.002, *P* = 0.005 ischemia-hypothermia slow rewarming vs. ischemia-normothermia, in the IGWM1, IGWM2, and PVWM respectively). There was an intermediate effect in the IGWM1 for ischemia-hypothermia fast rewarming and in the IGWM1 and IGWM2 in the ischemia-hypothermia slow rewarming groups, such that CC1+ mature oligodendrocyte numbers were significantly less than sham control in these areas (*P* = 0.01 ischemia-hypothermia fast rewarming vs. sham control, and *P* = 0.026, *P* = 0.042 ischemia-hypothermia slow rewarming vs. sham control, in the IGWM1 and IGWM2 respectively). There was no significant difference in numbers of CC1^+^ mature oligodendrocytes between the ischemia-hypothermia fast rewarming and ischemia-hypothermia slow rewarming groups (*P* = 0.975, *P* = 0.968, *P* = 1.000, in the IGWM1, IGWM2, and PVWM respectively). Further, there was no difference in numbers of cleaved caspase 3^+^ apoptotic cells between the groups (Supplementary Fig. 2, *P* = 0.456).

**Fig. 4:**
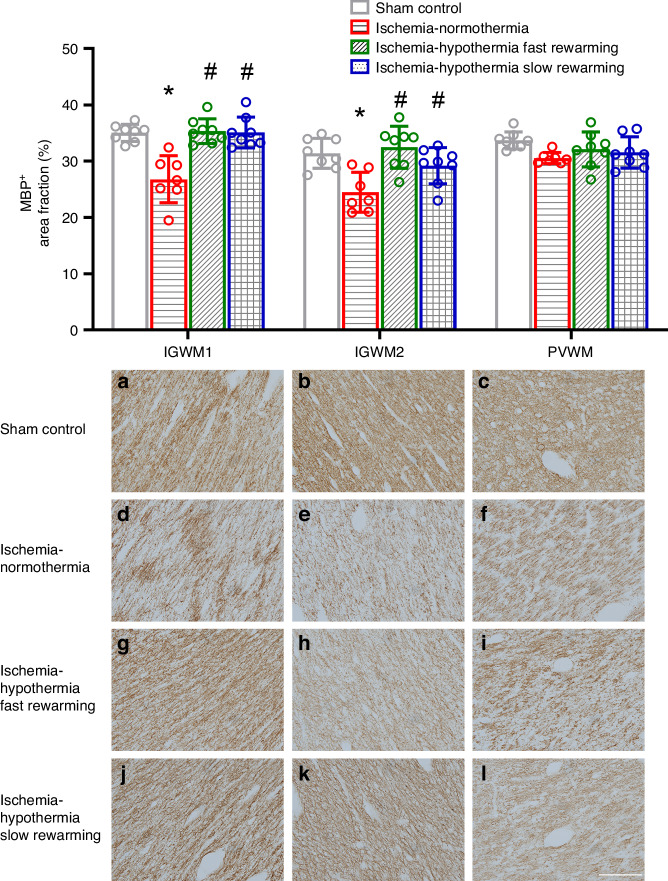
MBP^+^ area fraction and photomicrographs in the IGWM1, IGWM2 and PVWM in the sham control, ischemia-normothermia, ischemia-hypothermia fast rewarming and ischemia-hypothermia slow rewarming groups 7 days post HI. (Top) MBP^+^ area fraction within the IGWM1, IGWM2 and PVWM regions. (Bottom) representative photomicrographs of MBP^+^ in the IGWM1 (**a**, **d**, **g**, **j**), IGWM2 (**b**, **e**, **h**, **k**), PVWM (**c**, **f**, **i**, **l**). Scale bar in *L* = 100 µm. Sham control, *n* = 8; ischemia-normothermia, *n* = 7; ischemia-hypothermia fast rewarming, *n* = 8; and ischemia-hypothermia slow rewarming groups *n* = 8. Data are mean ± standard deviation. * *P* < 0.05 vs. sham control, # *P* < 0.05 vs. ischemia-normothermia.

Ischemia was associated with significantly reduced area fraction of MBP expression in the IGWM1 and IGWM2 compared with sham controls (Fig. 4, *P* < 0.001, *P* = 0.002 in the IGWM1 and IGWM2 respectively) but not in the PVWM (*P* = 0.062). Hypothermia was associated with a significant increase in MBP area fraction in both the IGWM1 and IGWM2 compared with ischemia-normothermia, to sham control levels (*P* < 0.001, *P* < 0.001 ischemia-hypothermia fast rewarming vs. ischemia-normothermia and *P* < 0.001, *P* = 0.048 ischemia-hypothermia slow rewarming vs. ischemia-normothermia, in the IGWM1 and IGWM2 respectively). There was no significant difference in MBP area fraction between ischemia-hypothermia fast rewarming and ischemia-hypothermia slow rewarming (*P* = 0.998, *P* = 0.218, in the IGWM1 and IGWM2 respectively).

**Fig. 5:**
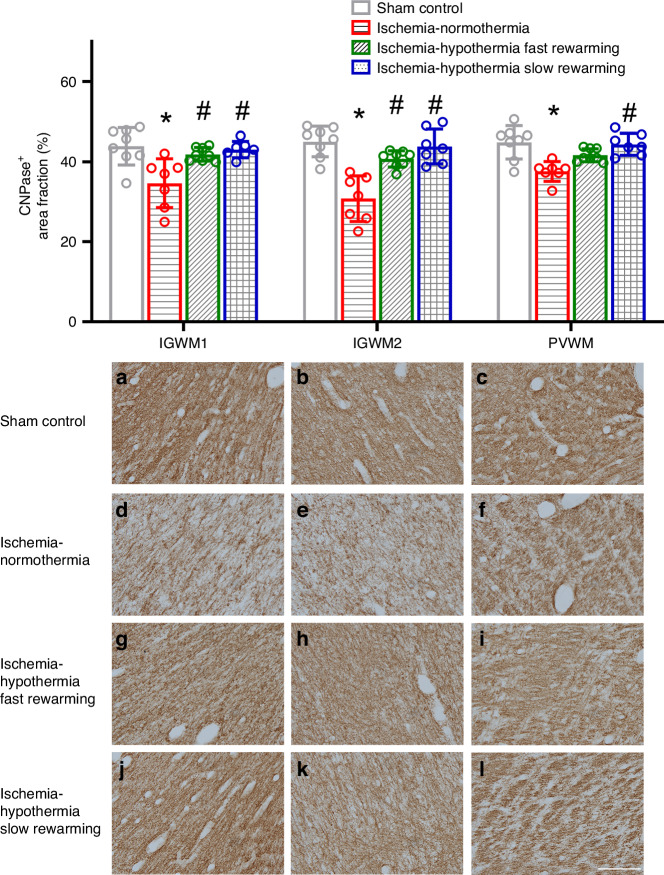
CNPase^+^ area fraction and photomicrographs in the IGWM1, IGWM2, and PVWM in the sham control, ischemia-normothermia, ischemia-hypothermia fast rewarming and ischemia-hypothermia slow rewarming groups 7 days post HI. (Top) CNPase^+^ area fraction within the IGWM1, IGWM2, and PVWM regions. (Bottom) representative photomicrographs of CNPase^+^ in the IGWM1 (**a**, **d**, **g**, **j**), IGWM2 (**b**, **e**, **h**, **k**), PVWM (**c**, **f**, **i**, **l**). Scale bar in *L* = 100 µm. Sham control, *n* = 8; ischemia-normothermia, *n* = 7; ischemia-hypothermia fast rewarming, *n* = 8; and ischemia-hypothermia slow rewarming groups *n* = 7. Data are mean ± standard deviation. * *P* < 0.05 vs. sham control, # *P* < 0.05 vs. ischemia-normothermia.

Ischemia was associated with a significant reduction in the area fraction of CNPase labeling in the IGWM1, IGWM2, and PVWM compared with sham control (Fig. 5, *P* < 0.001 for all). CNPase area fraction significantly increased in the ischemia-hypothermia fast rewarming group compared with ischemia-normothermia in the IGWM1 and IGWM2 (*P* = 0.009, *P* < 0.001 in the IGWM1 and IGWM2 respectively). CNPase area fraction significantly increased in the ischemia-hypothermia slow rewarming group compared with ischemia-normothermia in the IGWM1, IGWM2, and PVWM (*P* = 0.003, *P* < 0.001, *P* = 0.001, in the IGWM1, IGWM2 and PVWM respectively). There was no significant difference in CNPase area fraction between ischemia-hypothermia fast rewarming and ischemia-hypothermia slow rewarming (*P* = 0.942, *P* = 0.48, *P* = 0.291, in the IGWM1, IGWM2, and PVWM respectively).

**Fig. 6:**
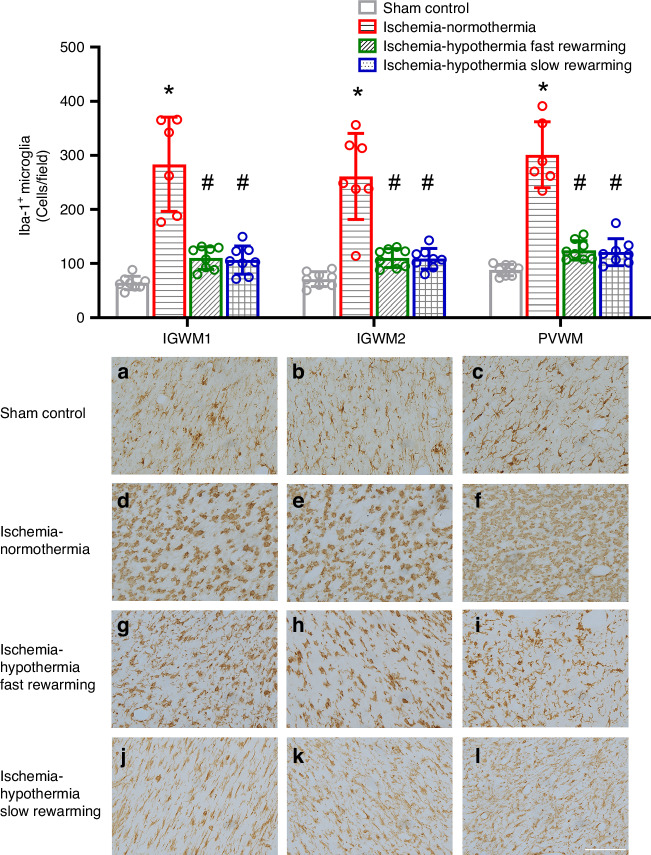
Iba-1^+^ microglia and photomicrographs in the IGWM1, IGWM2, and PVWM in the sham control, ischemia-normothermia, ischemia-hypothermia fast rewarming and ischemia-hypothermia slow rewarming groups 7 days post HI. (Top) Iba1^+^ microglia number within the IGWM1, IGWM2, and PVWM regions. (Bottom) representative photomicrographs of Iba1^+^ microglia in the IGWM1 (**a**, **d**, **g**, **j**), IGWM2 (**b**, **e**, **h**, **k**), PVWM (**c**, **f**, **i**, **l**). Scale bar in *L* = 100 µm. Sham control, *n* = 8; ischemia-normothermia, *n* = 7; ischemia-hypothermia fast rewarming, *n* = 8; and ischemia-hypothermia slow rewarming groups *n* = 8. Data are mean ± standard deviation. * *P* < 0.05 vs. sham control, # *P* < 0.05 vs. ischemia-normothermia.

Ischemia was associated with a significant increase in Iba-1^+^ microglia in all regions compared with sham control (Fig. 6, *P* < 0.001 for all regions). Hypothermia was associated with a significant reduction in the number of Iba-1^+^ microglia in the IGMW1, IGWM2 and PVWM compared with ischemia-normothermia (*P* < 0.001 for both ischemia-hypothermia fast rewarming vs. ischemia-normothermia and ischemia-hypothermia slow rewarming vs. ischemia-normothermia in all regions), back to sham control levels. There was no significant difference in the number of Iba-1^+^ microglia between ischemia-hypothermia fast rewarming and ischemia-hypothermia slow rewarming (*P* = 1.000 for all regions).

**Fig. 7:**
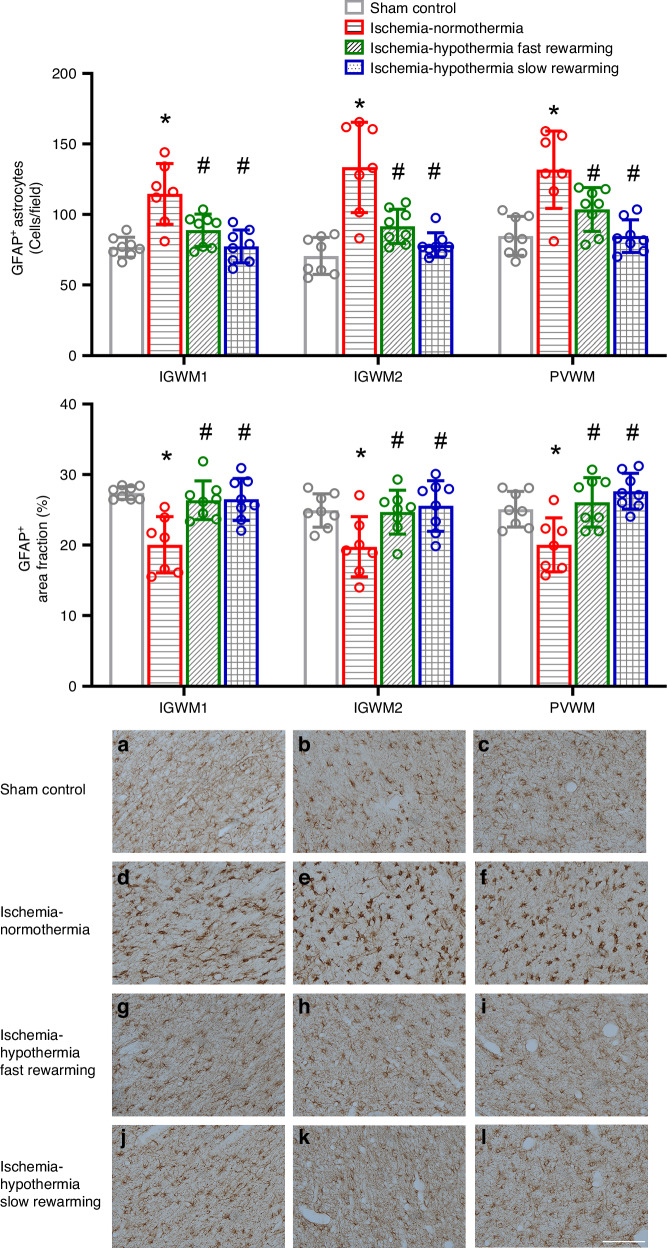
GFAP^+^ astrocyte number, area fraction, and photomicrographs in the IGWM1, IGWM2, and PVWM in the sham control, ischemia-normothermia, ischemia-hypothermia fast rewarming and ischemia-hypothermia slow rewarming groups 7 days post HI. (Top) GFAP^+^ astrocyte number within the IGWM1, IGWM2, and PVWM regions. (Middle) GFAP^+^ are fractions within the IGWM1, IGWM2, and PVWM regions. (Bottom) representative photomicrographs of GFAP^+^ astrocytes in the IGWM1 (**a**, **d**, **g**, **j**), IGWM2 (**b**, **e**, **h**, **k**), PVWM (**c**, **f**, **i**, **l**). Scale bar in *L* = 100 µm. Sham control, *n* = 8; ischemia-normothermia, *n* = 7; ischemia-hypothermia fast rewarming, *n* = 8; and ischemia-hypothermia slow rewarming groups *n* = 8. Data are mean ± standard deviation. * *P* < 0.05 vs. sham control, # *P* < 0.05 vs. ischemia-normothermia.

Ischemia was associated with a significant increase in the number of GFAP^+^ astrocytes in the IGWM1, IGWM2, and PVWM compared with sham control (Fig. 7, *P* < 0.001 for all regions). Hypothermia was associated with a significant reduction in the number of GFAP^+^ astrocytes in the IGWM1, IGWM2 and PVWM compared with ischemia-normothermia (*P* = 0.006, *P* < 0.001, *P* = 0.024 ischemia-hypothermia fast rewarming vs. ischemia-normothermia, and *P* < 0.001, *P* < 0.001, *P* < 0.001 ischemia-hypothermia slow rewarming vs. ischemia-normothermia, in the IGWM1, IGWM2 and PVWM respectively). There was no significant difference in the number of GFAP^+^ astrocytes between ischemia-hypothermia fast rewarming and ischemia-hypothermia slow rewarming (*P* = 0.348, *P* = 0.484, *P* = 0.168 in the IGWM1, IGWM2, and PVWM respectively).

Ischemia was associated with a decrease in the area fraction of GFAP in all regions compared with sham control (Fig. 7, *P* < 0.001, *P* = 0.030, *P* = 0.02 in the IGWM1, IGWM2, and PVWM respectively). Hypothermia was associated with a significant increase in GFAP area fraction compared with ischemia-normothermia (*P* = 0.001, *P* = 0.041, *P* = 0.005 ischemia-hypothermia fast rewarming vs. ischemia-normothermia, and *P* < 0.001, *P* = 0.013, *P* < 0.001 ischemia-hypothermia slow rewarming vs. ischemia-normothermia, in the IGWM1, IGWM2, and PVWM respectively). There was no significant difference in the area fraction of GFAP between ischemia-hypothermia fast rewarming and ischemia-hypothermia slow rewarming (*P* = 1.0, *P* = 0.98, *P* = 0.74 in the IGWM1, IGWM2, and PVWM respectively).

## Discussion

The current study demonstrates that in near-term fetal sheep exposed to cerebral ischemia, slow controlled rewarming at ~0.5 °C/h, over 10 h, after 72 h of hypothermia, was not associated with improved white matter outcomes, compared with fast spontaneous rewarming within 1 h. Hypothermia is neuroprotective, at least in part, by reducing secondary inflammation, excitotoxin release, oxidative stress, and programmed cell death.^17^ It has been speculated that these processes could be reactivated during rapid rewarming back to normothermia, and so exacerbate white matter injury. However, there is no clinical, or preclinical evidence from studies using an optimal duration of hypothermia for whether the rate of rewarming improves the recovery of white matter injury after treatment with hypothermia.^15^ In the present study, we found no difference in survival of total or mature oligodendrocytes, expression of myelin proteins, or attenuation of microglial and astrocytic proliferation between the two rewarming protocols. Thus, these results suggest that following a clinically established duration of hypothermia, slower rewarming does not improve white matter recovery compared with faster, spontaneous rewarming.

In the current study, ischemia was associated with marked loss of oligodendrocytes after 7 days recovery. Hypothermia, from 3 to 72 h after ischemia, increased survival of both total and mature oligodendrocytes, consistent with previous studies in near-term fetal sheep.^26–28^ There was no difference in numbers of total or mature oligodendrocytes between slow and fast rewarming groups. Consistent with this, we have previously shown that after hypothermia for 48 h, there was no difference in total or mature oligodendrocyte numbers between slow rewarming over 24 h, compared with fast rewarming within 1 h.^18^ In addition to the rate of rewarming having no effect on oligodendrocyte survival, we have previously shown that survival is not dependent on the duration of hypothermia. For example, extending the duration of hypothermia from 48 to 72 h and from 72 to 120 h did not further improve total oligodendrocyte survival.^11,25^ In contrast, oligodendrocyte survival appears to be highly sensitive to delay before initiation of hypothermia. For example, hypothermia started 90 min after HI increased survival of myelinating oligodendrocytes, whereas there was no protection when hypothermia was delayed until 5.5 h after HI.^29^ Speculatively, this suggests that cell death processes may evolve rapidly in oligodendrocytes, such that the rate of rewarming 72 h after HI had little effect on oligodendrocyte survival.

In the current study we showed that there was no improvement in the expression of MBP or CNPase with slow rewarming compared with fast rewarming. This is highly consistent with the lack of difference in mature myelinating oligodendrocytes between the groups. Interestingly only the ischemia-hypothermia slow rewarming group had significantly improved CNPase expression in the PVWM compared with ischemia-normothermia, albeit it was not different from fast rewarming. The reasons for why there was no significant improvement in CNPase expression in the PVWM in the ischemia-hypothermia fast rewarming group is not known.

Ischemia was associated with increased numbers of microglia, which were attenuated with hypothermia, similar to previous studies.^26–28^ There was no difference in numbers of microglia between ischemia-hypothermia fast and slow rewarming. Previously we have shown that hypothermia for 72 h was associated with greater suppression of microglial numbers than 48 h of hypothermia.^25^ Conversely, continuing hypothermia to 120 h was associated with a loss of suppression of microglial numbers, compared with 72 h of hypothermia.^25^ Together, these data suggest that 72 h represents an approximately optimal duration, beyond which further cooling does not provide any additional benefit. This could explain why slow rewarming provided no additional suppression of numbers of microglia in the present study, despite essentially extending hypothermia by around 10 h with the slower rewarming protocol.

In the present study, ischemia was associated with a significant increase in the number of GFAP^+^ astrocytes but decrease in area fraction of labeling at 7 days. This apparent contrast suggests that there was both proliferation of astrocytes as well as a morphological change associated with astrocytic activation^30^ consistent with changes seen in neonatal pigs 3 days after HI.^31^ Hypothermia restored GFAP^+^ astrocyte number and GFAP area fraction to sham control level. The rate of rewarming had no effect on numbers of GFAP^+^ astrocytes or GFAP area fraction. This is consistent with our previous findings that the rate of rewarming after 48 h of hypothermia had no effect on GFAP^+^ astrocyte number and GFAP area fraction.^18^

The slow rate of rewarming (over nearly 10 h) in the current study is consistent with the rates used in randomized control trials for therapeutic hypothermia of no greater than 0.5 °C/h.^32–34^ This cautious rate of rewarming was adopted due to concerns that faster rewarming could destabilize cardiovascular function and induce rebound seizures.^35,36^ It is important to consider, however, the practical implications of slower rewarming, including further delay in oral feeding and parental interaction, longer periods in a tertiary care unit, and for some infants a greater exposure to sedatives and analgesics. Together these practical considerations alongside the results of the current study suggest that slower rewarming is not an important contributor to recovery of white matter injury after HI. A very large randomized clinical trial testing differing rates of rewarming after hypothermia, would be necessary to confirm the present findings.

The current study has focused on the effect of the rate of rewarming on white matter injury. However, it is important to appreciate that HI injury affects many gray matter areas such as the cortex, hippocampus and basal-ganglia thalamus.^37^ Moreover, it remains unclear whether faster rewarming increases the risk of rebound seizures or recovery of EEG activity.^38,39^ Future studies will evaluate the effect of the rate of rewarming on gray matter, and recovery of EEG activity.

Overall, the present study has shown that slower rewarming did not provide any additional benefit for white matter injury compared with fast, spontaneous rewarming after 72 h of hypothermia. Slower rewarming was not associated with greater survival of oligodendrocytes, suppression of astrocytic and microglial proliferation or any increase in myelin expression. Overall, this shows that the rate of rewarming after HI with 72 h of hypothermia does not seem to be important for improving white matter injury after 7 days recovery.

## Supplementary information

Supplementary Figures

## Data Availability

The datasets generated during and/or analyzed during the current study are available from the corresponding author on reasonable request.
