# Supplementary material for: Slow rewarming after hypothermia does not ameliorate white matter injury after hypoxia-ischemia in near-term fetal sheep
Source: Pediatr Res. 2024 Aug 5;97(3):1209–19. doi: 10.1038/s41390-024-03332-y (PMC12055593; doi:10.1038/s41390-024-03332-y)
Supplement: Supplementary file 1 — Supplementary Figures [file 41390_2024_3332_MOESM1_ESM.pdf]

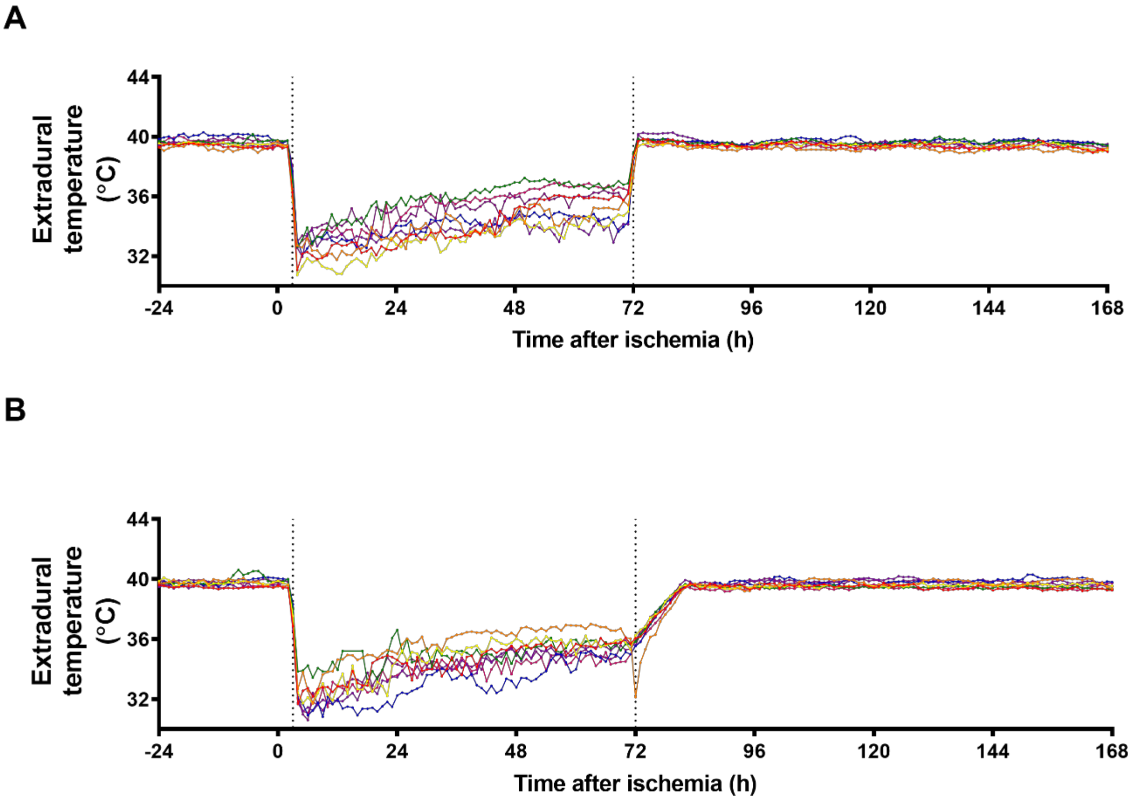

Supplementary Figure 1: Extradural temperature for individual animals in the ischemia-hypothermia fast rewarming (A) and ischemia-hypothermia slow rewarming (B) groups. Data shown is the hour average of extradural temperature. Dotted lines indicated the period when hypothermia was initiated and when rewarming began.

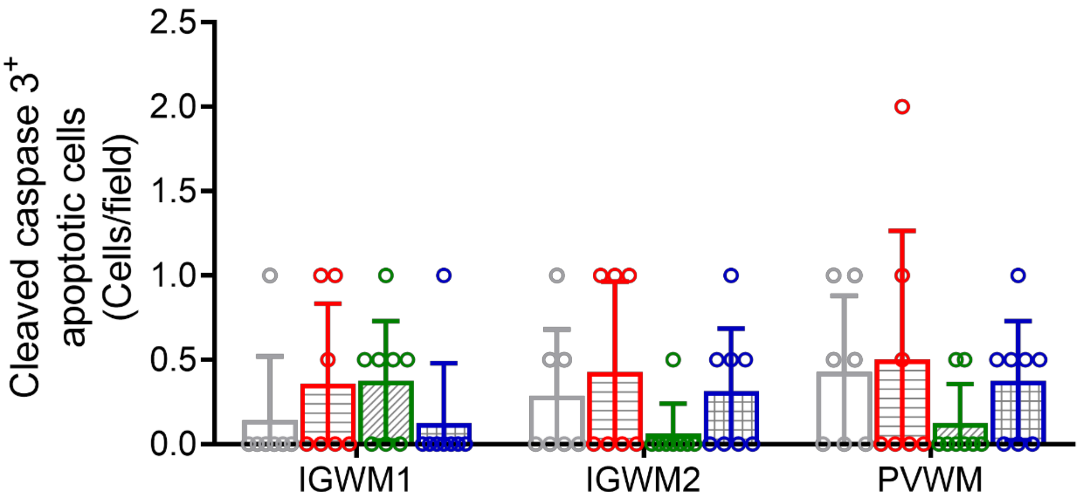

Supplementary Figure 2: Cleaved caspase 3+ apoptotic cell number in the IGWM1, IGWM2 and PVWM in the sham control, ischemia-normothermia, ischemia-hypothermia fast rewarming and ischemia-hypothermia slow rewarming groups 7 days post HI. Sham control, n=8; ischemia-normothermia, n=7; ischemia-hypothermia fast rewarming, n=7; and ischemia-hypothermia slow rewarming groups n=8**.** Data are mean ± standard deviation.
